# FGF23 promotes atrial fibrillation susceptibility through ETV1‐dependent sodium channel regulation and calcium handling abnormalities

**DOI:** 10.1113/EP093891

**Published:** 2026-08-24

**Authors:** Qiaoting Yu, Linwen Zeng, Zhijie Chen, Zhuhui Lin, Congying Wang, Lan Wang, Huixian Fu, Jiancheng Zhang

**Affiliations:** ^1^ Shengli Clinical Medical College Fujian Medical University Fuzhou Fujian PR China; ^2^ Department of Cardiology Shengli Clinical Medicine College of Fujian Medical University Fuzhou University Affiliated Provincial Hospital Fujian Provincial Hospital Fuzhou Fujian PR China; ^3^ Department of Cardiology Changji Prefecture People's Hospital in Xinjiang Uygur Autonomous Region Changji Xinjiang PR China

**Keywords:** atrial fibrillation, calcium homeostasis, ETV1, fibroblast growth factor 23, sodium current

## Abstract

Fibroblast growth factor 23 (FGF23) is closely associated with atrial fibrillation (AF), yet whether it modulates sodium and calcium currents in atrial myocytes remains unclear. ETV1, a key transcription factor regulating atrial electrical conduction, might also be involved in FGF23‐mediated AF. In this study, FGF23 intervention models were established via tail vein injection in mice and direct administration in vitro. ETV1 knockdown and overexpression were achieved in vitro using lentiviral vectors, and ETV1 knockout mice were generated. Patch‐clamp electrophysiology, calcium imaging and western blotting were performed to assess electrophysiological properties and AF susceptibility. Functionally, FGF23 increased both peak and residual sodium current measured 3 ms after peak activation, while also enhancing L‐type calcium current, prolonging action potential duration and promoting early and delayed after‐depolarizations. Consistent with these electrophysiological changes, Nav1.5 and RyR2 expression levels were upregulated, accompanied by increased intracellular calcium transients, thereby leading to increased AF susceptibility. In mechanistic analyses, ETV1 knockdown attenuated FGF23‐induced increases in sodium current and action potential parameters but did not reduce AF susceptibility, whereas ETV1 knockout increased AF inducibility. These findings suggest that FGF23 promotes AF by inducing coordinated electrical and calcium handling abnormalities that facilitate triggered activity, while ETV1 contributes to FGF23‐induced sodium channel regulation.

## Abstract (highlights)

- **What is the central question of this study?**
  Fibroblast growth factor 23 (FGF23) is associated with atrial fibrillation (AF), but whether it directly modulates sodium and calcium currents in atrial myocytes and whether ETV1 acts as a downstream mediator remain unclear.
- **What is the main finding and its importance?**
  Using in vivo and in vitro models with electrophysiology and calcium imaging, we show that FGF23 increases sodium and calcium currents, disrupts calcium handling and promotes susceptibility to AF. ETV1 is required for FGF23‐induced sodium channel regulation but does not determine AF susceptibility, identifying ETV1 as a key downstream mediator linking FGF23 to sodium channel remodelling in AF.

## 1 INTRODUCTION

Atrial fibrillation (AF) is one of the most common cardiac arrhythmias, characterized by high prevalence and frequent association with adverse cardiovascular and cerebrovascular events such as stroke, heart failure, cognitive decline and dementia. As such, it represents a major global public health challenge (Joglar et al., 2024; Li et al., 2022; Tsao et al., 2023). Electrical remodelling is a key mechanism underlying AF pathogenesis. The voltage‐gated sodium channel 1.5 (Nav1.5) serves as the primary sodium channel in atrial myocytes, and the peak sodium current (*I*_Na_) it mediates is essential for maintaining cellular excitability and conduction. Alterations in Nav1.5 channel activity and expression can lead to changes in atrial effective refractory period and action potential propagation velocity, thereby contributing to AF (Hayashi et al., 2015; Musa et al., 2015). Dysregulated calcium handling constitutes another crucial factor promoting electrical remodelling. Increased sarcoplasmic reticulum (SR) calcium leak, spontaneous calcium sparks and waves, and altered L‐type calcium current (*I*_Ca,L_) can trigger atrial ectopic activity and re‐entry, thereby increasing AF susceptibility (Mason et al., 2020; Nattel & Dobrev, 2012). Thus, atrial electrical remodelling resulting from disordered sodium and calcium handling is instrumental in AF development. However, the upstream molecular regulators governing sodium and calcium homeostasis in atrial myocytes remain poorly understood.

Fibroblast growth factor 23 (FGF23), an endocrine member of the FGF family, plays a central role in regulating phosphate and calcium homeostasis. Recent studies have indicated a correlation between elevated serum FGF23 levels and AF incidence (Mathew et al., 2014; Patel et al., 2020; Tan et al., 2022). Combining FGF23 levels with morphometric parameters (e.g., age, sex and body mass index) can reliably identify AF patients (Chua et al., 2021). Dong et al. (2019) and Lee et al. (2021) demonstrated that increased FGF23 activates atrial fibroblasts and promotes extracellular collagen deposition via STAT3 and Smad3 signalling pathways, leading to structural remodelling. Furthermore, FGF23 has been shown to enhance late sodium current (*I*_Na,L_) in atrial myocytes, facilitating atrial arrhythmogenesis (Huang et al., 2016). Additionally, Touchberry et al. (2013) identified FGF23 as a novel intracellular calcium modulator that acts through phospholipase C‐mediated calcium signalling (Lee et al., 2021). Although these findings underscore a strong association between circulating FGF23 and AF, the precise mechanisms remain incompletely elucidated. Given that current research has focused primarily on structural remodelling, we aimed to investigate whether FGF23 contributes to AF by perturbing sodium and calcium handling in atrial myocytes.

ETV1, a member of the ETS transcription factor family, is a key regulator of electrical conduction in both atria and ventricles (Monté et al., 1995; Shekhar et al., 2016). Recent evidence shows that ETV1 expression is significantly upregulated in the atria of patients with permanent AF, and cardiac‐specific overexpression of ETV1 in mice induces atrial arrhythmias accompanied by atrial enlargement, increased mass and interstitial fibrosis (Fatkin, 2018; Rommel et al., 2018). Moreover, ETV1 has been implicated in the transcriptional regulation of the Nav1.5 channel gene (Shekhar et al., 2016), yet its specific effects on sodium and calcium handling in atrial myocytes remain unclear. Members of the FGF family are known to drive ETV1 transcription in mammals (Chen et al., 1999); however, it is unknown whether FGF23 interacts with ETV1 and whether ETV1 participates in FGF23‐mediated AF promotion.

Therefore, the aim of this study was to investigate the roles of FGF23 and ETV1 in regulating sodium and calcium currents in atrial myocytes and to elucidate the functional role of ETV1 in FGF23‐induced AF. We sought to provide deeper insights into the pathogenesis of AF and identify potential therapeutic targets for its clinical prevention and treatment.

## 2 MATERIALS AND METHODS

### 2.1 Ethical approval

All animal study procedures and experiments were reviewed and approved by the Animal Welfare and Ethics Committee of Fujian Provincial Hospital (IACUC‐FPH‐SL‐20240304[0173]) on 5 March 2024, and all procedures conformed to the guidelines of Directive 2010/63/EU of the European Parliament on the protection of animals used for scientific purposes and to the US National Institutes of Health (NIH) *Guide for the Care and Use of Laboratory Animals*. All animals were fed a standard chow diet, with free access to water.

### 2.2 Experimental animals

All C57BL/6J mice were obtained from SPF (Beijing) Biotechnology Co., Ltd and housed in the Laboratory Animal Centre of the Chinese People's Liberation Army General Hospital. The mice were maintained under a standard 12 h–12 h light–dark cycle (lights on from 08:00 to 20:00 h), with ad libitum access to food and water. No animals died before the completion of the experiments. All were included in the subsequent analyses. ETV1 knockout (ETV1‐KO) mice were purchased from Cyagen (Suzhou) Biosciences Co., Ltd. All experimental mice used in this study were male. FGF23 administration was performed as described in previous studies (Faul et al., 2011). Briefly, recombinant FGF23 was dissolved in PBS at a dose of 40 µg/kg in a final volume of 200 µL and injected into the tail vein as a single bolus injection twice daily with an 8 h interval between injections, for 5 days consecutively, as previously described in studies investigating the cardiac effects of FGF23 (Faul et al., 2011). Control mice received an equal volume of PBS using the same injection protocol. Mice were randomly assigned to experimental groups. Investigators were blinded to group allocation during data acquisition and analysis where applicable. Animals were monitored throughout the study, and humane endpoints were applied when necessary to minimize suffering.

### 2.3 Isolation, culture and transfection of primary atrial myocytes from neonatal mice

Neonatal C57/BL6J mice aged 1–2 days were killed by decapitation. The hearts were rapidly excised, and the atria were carefully dissected and transferred to ice‐cold Hanks' Balanced Salt Solution (HBSS) solution (Gibco, USA) without Ca^2+^ and Mg^2+^. The atrial tissue was then minced three to five times rapidly using a fresh surgical blade and digested in an enzymatic solution consisting of 0.08% trypsin (Gibco, USA) and 0.001 mg/mL collagenase II (Worthington, USA) in HBSS. The digestion was performed in a stepwise manner, with the supernatant being collected every 2 min and transferred into complete culture medium [high‐glucose Dulbecco's modified Eagle's medium (Gibco, USA) supplemented with 10% fetal bovine serum and 1% penicillin–streptomycin] to terminate enzymatic activity. This process was repeated until all tissue fragments were fully digested. The collected supernatants were filtered to remove debris and undigested collagen, followed by centrifugation at 376 × *g* for 15 min. The pellet was resuspended in complete medium and subjected to differential plating for 2 h to reduce non‐cardiomyocyte contamination. After this preplating step, the cell suspension was gently aspirated, triturated uniformly and seeded into appropriate culture plates or dishes. Cells were maintained in a humidified incubator at 37°C for further culture.

The ETV1 overexpression or knockdown lentivirus and its negative control were constructed by Genechem Co., Ltd (Shanghai, China). Primary myocytes were infected with the lentivirus at a multiplicity of infection (MOI) of 30, in the presence of 8 µg/mL Polybrene to enhance transduction efficiency. Approximately 4–5 days postinfection, successfully transduced cells were selected using puromycin to establish stable cell lines.

Cells in the FGF23 intervention group were treated with 25 ng/mL FGF23 for 48 h, whereas the control group received an equal volume of PBS.

### 2.4 Intra‐oesophageal burst pacing and induction of AF

All experiments were performed as previously described (Chen et al., 2019). Briefly, mice were anaesthetized with inhaled isoflurane (1.5%–2% in oxygen) and instrumented with subcutaneous electrodes for ECG recording (Power Lab 16/35, AD Instruments, Castle Hill, NSW, Australia). Intra‐oesophageal burst pacing was used to assess susceptibility to AF. The electrode was inserted through the oesophagus and placed at the site with the lowest threshold for atrial capture. Atrial pacing was performed at twice the diastolic threshold value using two poles on the pacing catheter. First, the pulse was paced for 10 s with a cycle length of 100 ms, followed by burst stimulation for 10 s with a cycle length of 30 ms until 10 consecutive burst stimulations or AF were induced. AF was defined as irregular, rapid atrial activation, with varying electrogram morphology lasting ≥10 s. All mice were allowed 3 min of recovery in sinus rhythm between stimulations for respiratory and circulatory recovery. The occurrence and duration of AF in each group were observed and recorded.

### 2.5 Adult atrial myocyte isolation

Isolated mouse hearts were perfused using a Langendorff system. Mice were injected intraperitoneally with 125 units of heparin 20 min prior to cervical dislocation. The chest was opened, and the heart was rapidly excised and positioned on the perfusion needle. Cervical dislocation was subsequently performed to ensure death. The heart was initially perfused for 3–5 min at a flow rate of 4 mL/min with a buffer solution (pH 7.0, 37°C) containing the following (mmol/L): 112.94 NaCl, 4.69 KCl, 1.21 KH_2_PO_4_, 1.16 Na_2_HPO_4_, 1.25 MgSO_4_, 11.9 NaHCO_3_, 10.00 KHCO_3_, 10.03 HEPES, 14.98 taurine, 18.00 glucose and 10.11 2,3‐Butanedione monoxime (BDM). Subsequently, enzymatic digestion was performed by perfusing the heart for 11–13 min with a digestion solution containing trypsin (Gibco, USA) and collagenase II (Worthington, USA). Following digestion, the atria were dissected, and digestion was terminated. The atrial tissue was filtered to isolate individual cardiomyocytes. A stepwise reintroduction of calcium was performed to avoid calcium shock. Initially, 10 µL of 100 mM CaCl_2_ was gently added to the cell suspension, followed by 5 min of incubation. Then, 20 µL of 100 mM CaCl_2_ was added, and the cells were incubated for another 5 min. This was repeated with a second addition of 20 µL of 100 mM CaCl_2_ and a final 5 min incubation. Throughout this gradual calcium reintroduction, atrial myocytes were observed to settle gently at the bottom of the tube. The cells were then used for subsequent electrophysiological experiments.

### 2.6 Patch clamp

Patch‐clamp experiments were used to record action potentials, *I*_Ca,L_ and *I*_Na_ in atrial myocytes. Membrane potential and membrane currents were recorded at a physiological temperature (35°C–37°C) using the patch‐clamp technique in whole‐cell recording mode. Temperature was controlled with a TC‐344C heater controller (Warner Instruments). A P70 horizontal puller (Sutter Instruments) was used to pull borosilicate glass pipettes (World Precision Instruments) to a resistance of 2–4 MΩ. Membrane potentials were sampled at 10 kHz using a 1440A Digidata (Axon Instruments) controlled by pCLAMP programs (v.10.2). Cell capacitance, series resistance (compensation, 70%–80%) and junction potentials were compensated using the circuitry of the Axon Multiclamp 700B Amplifier (Molecular Devices, USA) and low‐pass filtered at 5 kHz. Protocols, data acquisition, storage, analysis, current fitting and offline subtraction were performed using Clampfit v.10.4 (Molecular Devices), and all curves were fitted with Origin (Microcal software).

Action potentials were recorded after applying a 2.5 ms, 1 nA depolarizing stimulus in current‐clamp mode. Delayed after‐depolarizations (DADs) were defined as the presence of spontaneous depolarization of the impulse after full repolarization occurred. Early after‐depolarizations (EADs) were defined as depolarizations occurring during phase 2 or phase 3 of the action potential before full repolarization. Micropipettes were filled with a solution containing (mM): 20 KCl, 110 potassoi, aspartate, 1 MgCl_2_, 5 Mg_2_ATP, 10 HEPES, 0.5 EGTA, 0.1 NaGTP and 5 Na_2_ phosphocreatine, titrated to a pH of 7.2 with KOH. Cells were bathed in a solution that contained (mM): 140 NaCl, 1 CaCl_2_, 1 MgCl_2_, 10 HEPES, 4 KCl and 5 glucose, with pH adjusted to 7.36 with CsOH.

*I*_Ca,L_ was recorded in the voltage‐clamp mode from a holding potential of −40 mV. Depolarizing pulses were applied from −40mV to +50 mV in 10‐mV increments, with each pulse lasting 50 ms. These recordings were used to plot the current–voltage relationship (*I–V* curve), steady‐state activation (SSA) or steady‐state inactivation (SSI) curves. To record *I*_Ca,L_, the pipette solution contained (mM): 120 CsCl, 1 CaCl_2_, 5 MgCl_2_, 11 EGTA, 10 HEPES and 5 Na_2_ATP, adjusted to pH 7.2 with CsOH. The external solution contained (mM): 140 NaCl, 2 CaCl_2_, 1 MgCl_2_, 4 KCl, 10 HEPES and 10 glucose, adjusted to pH 7.4 with CsOH. Tetrodotoxin (5 µM) was added to block sodium current when recording calcium currents. For each cell, current amplitude data were normalized with respect to cell capacitance (current density, in picoamperes per picofarad), and the *I–V* curve was plotted. Voltage‐dependent activation and SSI profiles were fitted to a Boltzmann equation: *a* = 1/{1 + exp[−(*V*_m_ − *V*_1/2_)/*k*]}, where *a* is the normalized conductance, *V*_m_ is the test potential, *V*_1/2_ is the potential at which current is half‐activated/inactivated, and *k* is the slope factor. Electrophysiological data were analysed using Clampfit v.10.4 (Axon Instruments) and Origin (OriginLab Corporation).

*I*_Na_ was recorded in voltage‐clamp mode from a holding potential of −80 mV. Test pulses from −70 to +70 mV were applied in 10 mV increments with a duration of 50 ms to activate sodium channels fully. The recordings were used to analyse the *I–V* curve, SSA and SSI properties. To isolate *I*_Na_, the pipette solution contained (mM): 135 CsCl, 10 EGTA, 2 MgCl_2_, 10 HEPES and 4 Na_2_ATP, adjusted to pH 7.2 with CsOH. The extracellular solution contained (mM): 30 NaCl, 110 CsCl, 1.5 CaCl_2_, 1.2 MgCl_2_, 10 HEPES, 5 glucose and 0.2 CdCl_2_ (to block calcium currents), adjusted to pH 7.4 with CsOH. Tetrodotoxin (30 µM) was applied at the end of selected experiments to confirm *I*_Na_ identity. Current amplitudes were normalized to cell capacitance to obtain current density (in picoamperes per picofarad). SSA and inactivation curves were fitted with the Boltzmann equation: *G*/*G*_max_ = 1/{1 + exp[−(*V* − *V*_1/2_)/*k*]}, where *G* is conductance, *V* is test potential, *V*_1/2_ is half‐activation/inactivation voltage, and *k* is the slope factor. All electrophysiological data were analysed using Clampfit v.10.4 (Axon Instruments) and Origin (OriginLab Corporation).

### 2.7 Ca^2+^ imaging

Isolated atrial myocytes were incubated with 2 µmol/L fluo‐4 AM (Invitrogen, USA) for 20 min, then equilibrated in fresh Tyrode solution with 1.8 mM Ca^2+^ containing 250 µmol/L probenecid (Sigma, USA) for 20 min to allow dye de‐esterification in laminin‐coated dishes. The Myocyte Calcium & Contractility Recording System (IonOptix, Westwood, MA, USA) was used to record transient changes in Ca^2+^ concentrations. Calcium transient parameters were analysed using IonWizard v.6.5 software. Fluorescence signals were normalized to baseline fluorescence (*F*_0_), defined as the average fluorescence recorded immediately before stimulation, and expressed as *F*/*F*_0_. Calcium transient amplitude was defined as peak *F*/*F*_0_. Time to 50% peak (half‐decay time) and the decay time constant (τ) were derived from the fluorescence decay phase, with τ obtained by mono‐exponential fitting.

Atrial myocytes were preconditioned by field stimulating at 1 Hz to achieve a steady state of ≥20 beats, and Ca^2+^ sparks were acquired over a 10 s rest period. For Ca^2+^ spark recordings, atrial myocytes were imaged using a laser‐scanning confocal microscope (SP5, Leica Microsystems) operated in line‐scan mode (332 Hz, 0.120 µm per pixel). Ca^2+^ spark analysis was performed using SparkMaster plugin in ImageJ. Events were defined as Ca^2+^ sparks when fluorescence intensity exceeded 3.8 times the SD of baseline noise, measured in regions without spontaneous Ca^2+^ release events.

### 2.8 Western blot

Primary atrial myocytes or atrial tissues were lysed on ice for 45 min using a strong RIPA lysis buffer supplemented with 1 mM phenylmethylsulphonyl fluoride. Protein lysates were then clarified by centrifugation at 13,528 × *g* for 15 min at 4°C. Protein concentrations were determined using the bicinchoninic acid (BCA) assay (Beyotime Biotech, China), adjusted accordingly, and mixed with 5× protein loading buffer (Beyotime Biotech, China), followed by boiling at 100°C for 10 min in a metal bath. Proteins were separated by SDS‐PAGE and transferred onto polyvinylidene fluoride membranes (Millipore Corporation, Billerica, MA, USA). The membranes were blocked with 5% non‐fat milk for 1 h at room temperature and incubated overnight at 4°C with primary antibodies selected according to the molecular weights of the target proteins. Primary antibodies used included: Nav1.5 (Abcam, USA, 1:1000), Cav1.2 (Abcam, USA, 1:1000), RyR2 (Abcam, USA, 1:1500), NCX1.1 (Abcam, USA, 1:1000), CAMKII (Abcam, USA, 1:1000), SERCA2a (Abcam, USA, 1:1000) and GAPDH (Abcam, USA, 1:3000). The membranes were then incubated for 1 h at room temperature with horseradish peroxidase‐conjugated secondary antibodies: goat anti‐rabbit IgG (Proteintech Group, USA) or goat anti‐mouse IgG (Proteintech Group, USA). Protein expression was detected using enhanced chemiluminescence substrate (MedChemExpress, USA). Band intensities were quantified using ImageJ software.

### 2.9 Statistical analysis

Patch‐clamp current data were analysed using Origin Pro 8. ECG and intra‐oesophageal stimulation physiological signal data were processed using LabChart v.8. Statistical graphs were plotted using GraphPad Prism v.9. Data distribution was assessed using the Shapiro–Wilk normality test. Normally distributed continuous variables were presented as the mean ± SD. Comparisons between two groups were performed using Student's unpaired *t*‐test, or Welch's *t*‐test when variances were unequal. Non‐normally distributed data were presented as the median (interquartile range) and analysed using the Mann–Whitney *U*‐test. For comparisons among three or more groups, one‐way ANOVA followed by Tukey's multiple‐comparisons test was used for normally distributed data. Categorical variables were expressed as percentages and analysed using the χ^2^ test. Fisher's exact test was applied when the expected cell count was less than five. Significance levels are indicated as ns (*P* > 0.05), *P < 0.05, **P < 0.01, ***P < 0.001 and ****P < 0.0001. For electrophysiological experiments, cardiomyocytes were isolated from adult mice or from neonatal atrial cultures. The number of cells recorded varied between experiments and is reported in each figure legend together with the number of animals (biological replicates). All data were obtained from at least three independent biological replicates. Individual data points shown in figures represent single cells, and the number of animals and cells (*n*) are specified in each figure legend.

## 3 RESULTS

### 3.1 Elevated FGF23 levels increase susceptibility to AF in mice

To investigate the impact of elevated FGF23 levels on AF susceptibility, we established a murine model by administering FGF23 via tail vein injection to simulate systemic FGF23 overexpression. Compared with the control group, FGF23‐treated mice exhibited a significantly higher AF inducibility rate (Figure 1a,b) and prolonged AF duration (38.28 ± 2.77 s vs. 9.88 ± 2.45 s, *P* < 0.01; Figure 1c).

**FIGURE 1:**
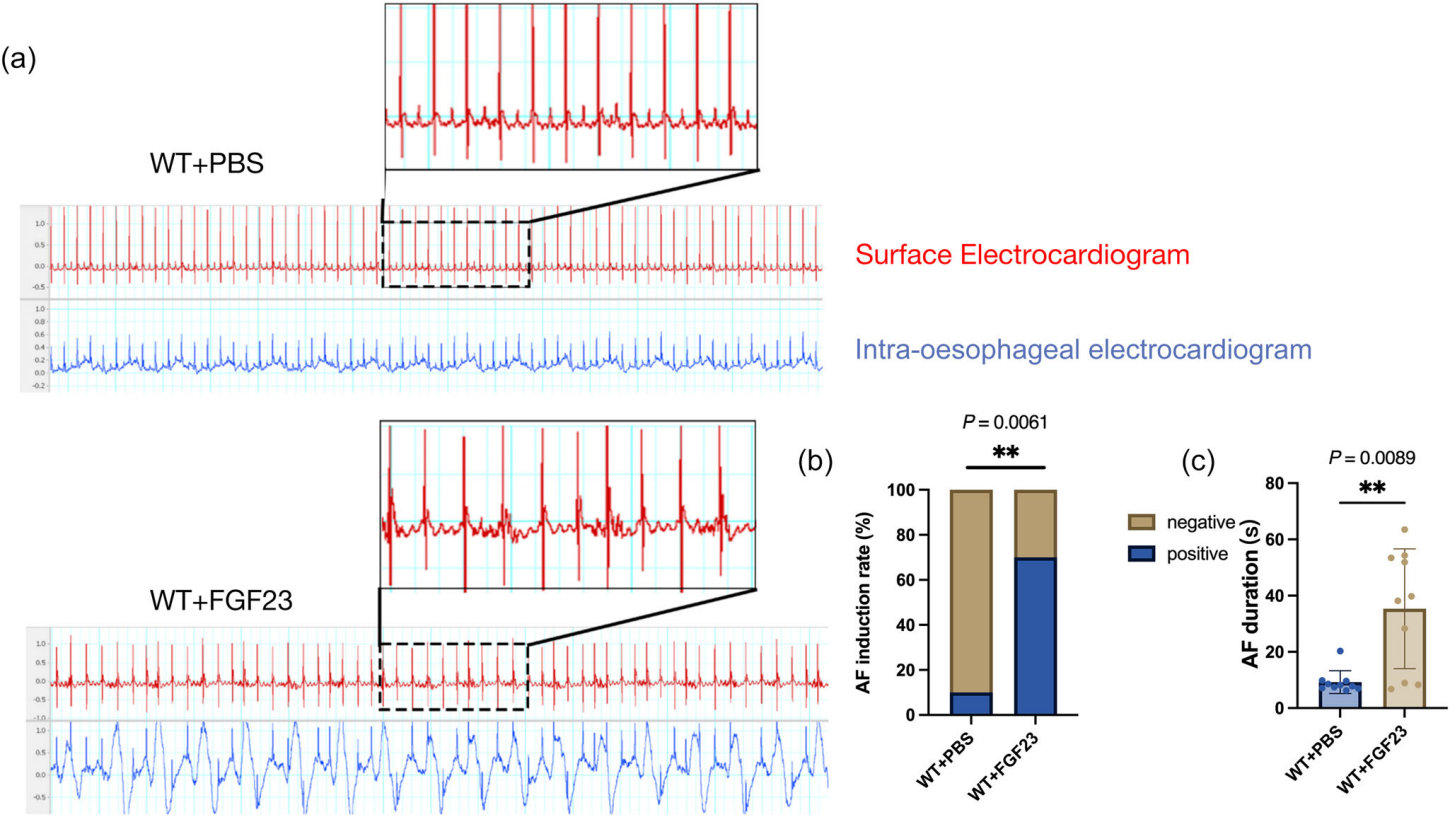
Effect of FGF23 administration on atrial fibrillation susceptibility in mice. (a) Representative surface and intra‐oesophageal ECGs illustrating AF induction after atrial pacing in PBS and FGF23 groups. (b, c) Quantitative analysis of AF induction rate and AF duration in each group. *n* = 10. **P < 0.01. Abbreviation: AF, atrial fibrillation. Abbreviations: AF, atrial fibrillation; ECG, electrocardiogram; PBS, phosphate‐buffered saline; WT, wild‐type.

To explore whether FGF23 modulates AF susceptibility through alterations in the electrophysiological properties of atrial myocytes, we isolated and compared atrial cardiomyocytes from both groups. Our results demonstrated that FGF23 significantly increased the action potential amplitude (APA) without affecting the resting membrane potential (RP) or maximum upstroke velocity (*V*_max_) (Figure 2a–d; Table 1). Moreover, FGF23 treatment markedly prolonged action potential duration (APD) at 20%, 50% and 90% repolarization (APD_20_, APD_50_ and APD_90_, respectively; Figure 2e–g; Table 1). Additionally, FGF23 enhanced the incidence of DADs and triggered activity (TA) (Figure 2h–i).

**FIGURE 2:**
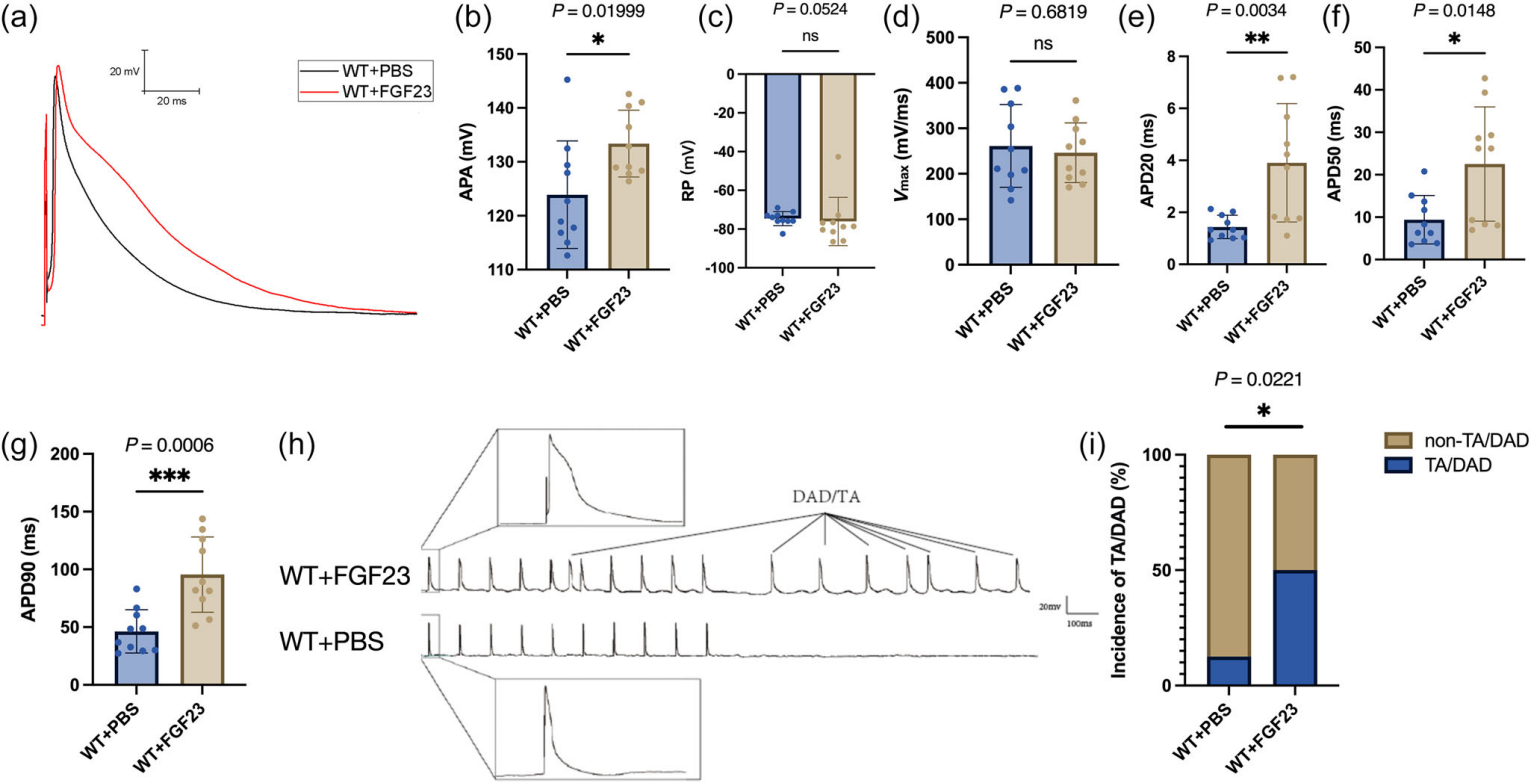
Effects of FGF23 on action potential properties and triggered activity in adult mouse atrial myocytes. (a) Typical action potential trajectories of the two groups of mice. (b–d) Quantitative analysis of the amplitude of APA (b), RP (c) and *V*_max_ (d) in each group. *n* = 10 cells from six mice. (e–g) Quantitative analysis of APD_20_ (e), APD_50_ (f) and APD_90_ (g) in each group. *n* = 10 cells from six mice. (h) Representative action potential traces from the WT+FGF23 and WT+PBS groups. The rectangular enlarged area shows a prolonged action potential in WT+FGF23 group, and the straight segment indicates an increased incidence of DAD/TA in the WT+FGF23 group. (i) Incidence of TA or DAD in each group. *n* = 16 cells from nine mice. *P < 0.05, **P < 0.01 and ***P < 0.001. Abbreviations: APA, amplitude of action potential; ns, not significant; RP, resting potential; *V*_max_, maximum rate of action potential rise. Abbreviations: APA, action potential amplitude; APD20, action potential duration at 20% repolarization; APD50, action potential duration at 50% repolarization; APD90, action potential duration at 90% repolarization; DAD, delayed afterdepolarization; PBS, phosphate‐buffered saline; RP, resting membrane potential; TA, triggered activity; Vmax, maximum upstroke velocity; WT, wild‐type.

**TABLE 1:** Action potential parameters in adult mouse atrial myocytes.

Parameter	WT+PBS	WT+FGF23	*P*‐value
RP, mV	−74.74 (−75.89, −72.44)	−78.72 (−82.55, −75.64)	0.0524
APA, mV	123.9 ± 9.97	133.4 ± 6.20	0.0200
*V*_max_, mV/ms	261.1 ± 91.04	246.3 ± 65.58	0.6819
APD_20_, ms	1.4 ± 0.45	3.9 ± 2.28	0.0035
APD_50_, ms	9.4 ± 5.73	22.51 ± 13.46	0.0148
APD_90_, ms	46.3 ± 18.72	95.5 ± 32.60	0.0006

Abbreviations: APA, action potential amplitude; APD20, action potential duration at 20% repolarization; APD50, action potential duration at 50% repolarization; APD90, action potential duration at 90% repolarization; FGF23, fibroblast growth factor 23; PBS, phosphate‐buffered saline; RP, resting membrane potential; Vmax, maximum upstroke velocity; WT, wild‐type.

### 3.2 FGF23 stimulation increases *I*_Na_ in murine atrial myocytes

*I*_Na_ was recorded from each group of cells in voltage‐clamp mode. The results revealed that FGF23 treatment significantly increased *I*_Na_ density at −30 mV compared with the control group, along with an elevated peak *I*_Na_ density (WT+PBS vs. WT+FGF23: −99.67 ± 35.71 vs. −167.4 ± 92.27 pA/pF, *P* < 0.05). No statistically significant differences were observed at other voltages (Figure 3a–c). Subsequently, Boltzmann distribution fitting was performed to determine the half‐activation voltage (*V*_1/2act_), slope factor of activation (*K*_act_), half‐inactivation voltage (*V*_1/2, inact_) and slope factor of inactivation (*K*_inact_). No significant differences were detected in *V*_1/2act_ (WT+FGF23 vs. WT+PBS: −45.91 ± 8.10 mV vs. −45.83 ± 7.90 mV) or *K*_act_ (WT+FGF23 vs. WT+PBS: 2.55 ± 1.74 vs. 1.55 ± 0.68 mV) between the two groups (Figure 3d–h). However, a rightward shift was observed in the SSI curve, accompanied by an increase in *V*_1/2inact_ (WT+PBS vs. WT+FGF23: −74.06 ± 4.94 vs. –63.4 ± 4.76 mV) and a decrease in *K*_inact_ (WT+PBS vs. WT+FGF23: 6.79 ± 3.14 vs. 2.25 ± 1.50 mV; Figure 3e), suggesting delayed *I*_Na_ inactivation following FGF23 administration. Furthermore, the recovery time constant (τ_1_) of *I*_Na_ was calculated, and no significant difference was observed between the two groups (WT+PBS vs. WT+FGF23: −2.36 ± 0.33 vs. –2.49 ± 0.20 ms; Figure 3f,i). Additionally, no significant difference was observed in the residual sodium current measured 3 ms after peak *I*_Na_ between the WT+PBS and WT+FGF23 groups [5.33% (3.51%–8.48%) vs. 6.76% (5.85%–8.21%), respectively; Figure 3j,k]. Superimposition of the SSA and inactivation curves indicated a markedly enhanced window current in FGF23‐treated mice (Figure 3l). Western blot analysis also demonstrated that FGF23 treatment significantly upregulated Nav1.5 protein expression in mouse atrial tissue (Figure 3m,n).

**FIGURE 3:**
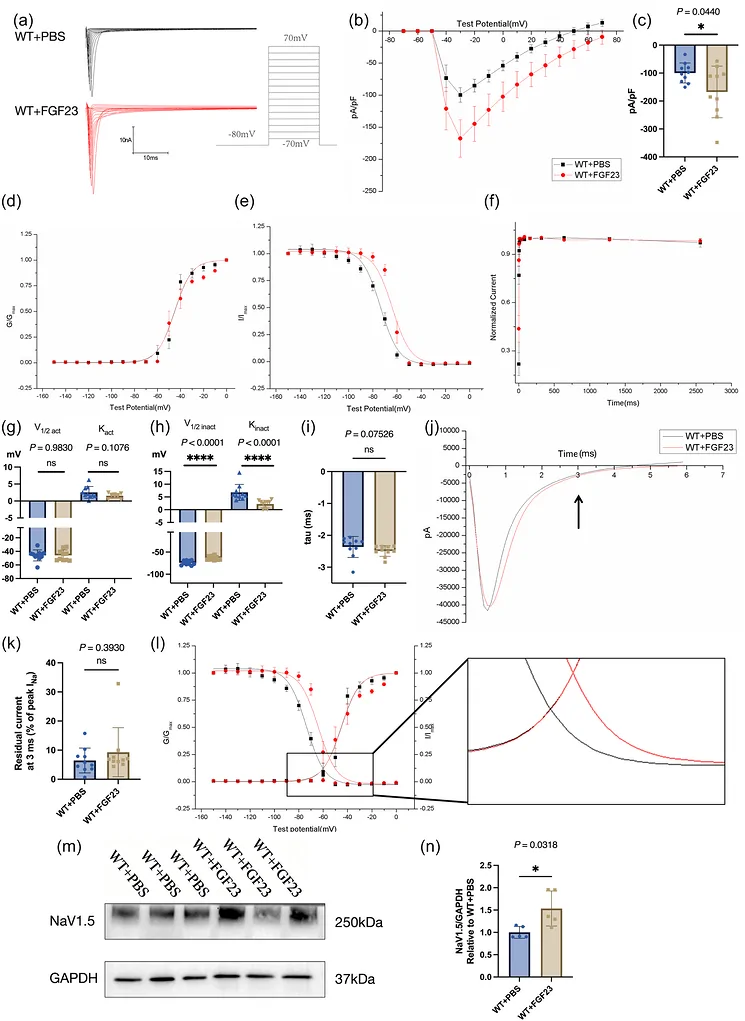
Effect of FGF23 on sodium current (*I*_Na_) in adult mouse atrial myocytes. (a) Typical *I*_Na_ trajectories of acutely isolated mouse atrial myocytes in voltage‐clamp mode, with stimulation procedure on the right. (b, c) *I–V* curve of *I*_Na_ and quantitative analysis of peak sodium current density at −30 mV in PBS and FGF23 groups. *n* = 10 cells from 6 mice. (d–i) Steady‐state gating and recovery kinetics of *I*_Na_: (d) activation; (e) inactivation; and (f) recovery curves of *I*_Na_. Corresponding parameter statistics are shown as follows: (g) *V*_1/2 act_ and *K*_act_; (h) *V*_1/2 inact_ and *K*_inact_; and (i) recovery τ, *n* = 10. (j, k) Residual sodium current measured 3 ms after peak *I*_Na_ at a test potential of −30 mV in atrial myocytes from both groups (black arrow indicates the current measured 3 ms after the peak current), *n* = 10 cells from six mice. (l) Window currents of sodium channels (black line indicates WT+PBS group; red line indicates WT+FGF23 group). (m, n) Representative western blotting and quantitative analysis of Nav1.5 proteins in atria of PBS or FGF23 mice. *n* = 5. ns, not significant, *P < 0.05 and ****P < 0.0001. Abbreviations: *I*_Na_, sodium current; NaV1.5, voltage‐gated sodium channel 1.5; PBS, phosphate‐buffered saline; WT, wild‐type.

### 3.3 FGF23 stimulation also increased *I*_Na_ in primary atrial myocytes, prolonged APD and enhanced the incidence of EADs

We examined the electrophysiological effects of FGF23 on APD and EADs in neonatal mouse atrial myocytes. Consistent with previous findings, cells treated with FGF23 showed significantly larger APA and *V*_max_, although no significant difference was detected in RP (Figure 4a–d; Table 2). Meanwhile, APD_20_, APD_50_ and APD_90_ were all significantly prolonged in the FGF23 group (Figure 4e–g; Table 2). Notably, a significantly higher incidence of EADs was observed in FGF23‐treated atrial myocytes (Figure 4h,i).

**FIGURE 4:**
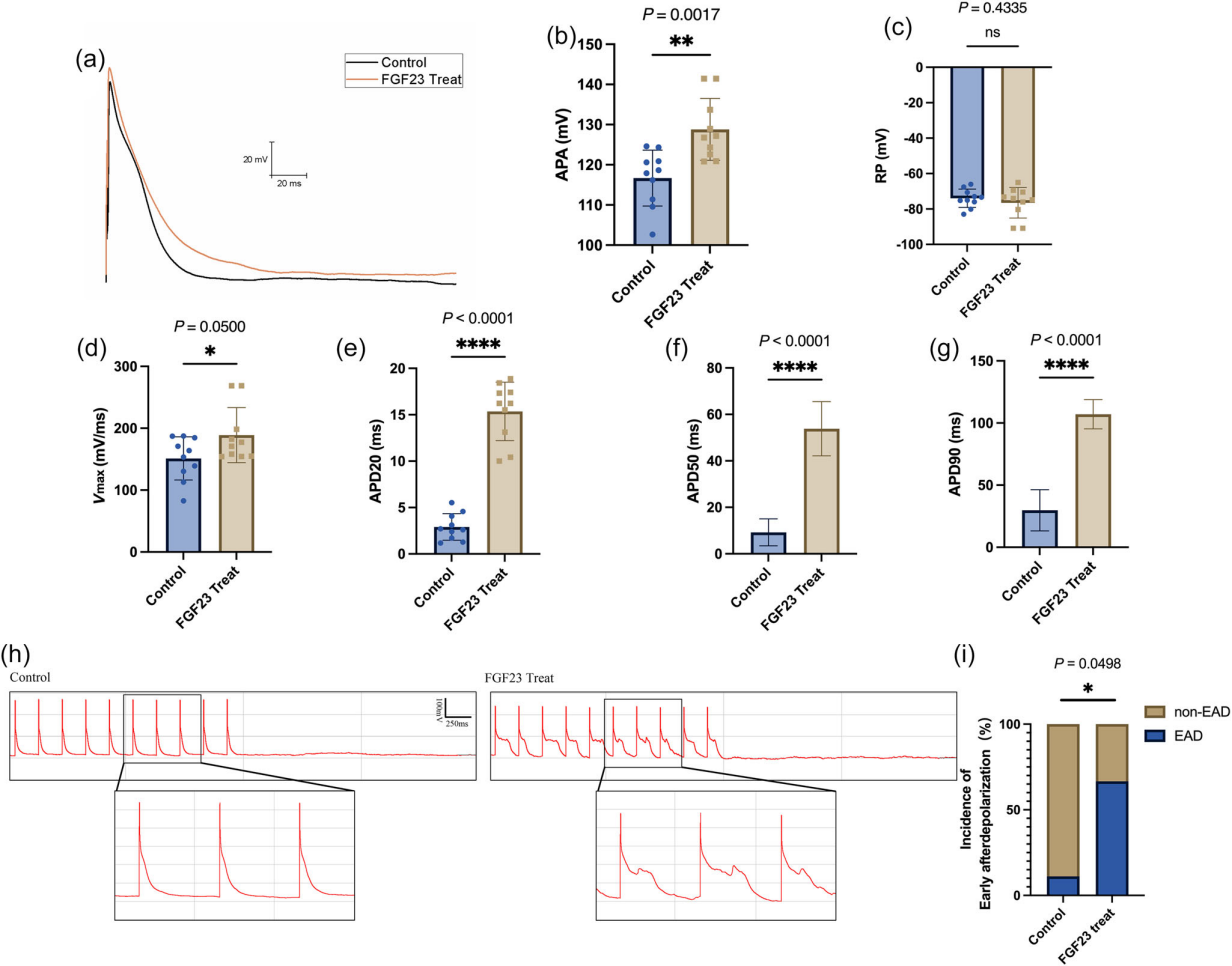
Effects of FGF23 stimulation on action potential properties and EAD incidence in primary atrial myocytes. (a) Typical action potential trajectories of the two groups of primary atrial myocytes. (b–d) Quantitative analysis of APA (b), RP (c) and *V*_max_ (d) in each group. *n* = 10 cells from six mice. (e–g) Quantitative analysis of APD_20_ (e), APD_50_ (f) and APD_90_ (g) in each group. *n* = 10 cells from six mice. (h) Typical action potential trajectories of primary atrial myocytes in each group (the zoom‐in view shows EAD). (i) Statistics of the incidence of EAD in each group. *n* = 10 cells from six mice. ns, not significant, *P < 0.05, **P < 0.01 and ****P < 0.0001. Abbreviations: APA, action potential amplitude; APD20, action potential duration at 20% repolarization; APD50, action potential duration at 50% repolarization; APD90, action potential duration at 90% repolarization; EAD, early afterdepolarization; PBS, phosphate‐buffered saline; RP, resting membrane potential; Vmax, maximum upstroke velocity; WT, wild‐type.

**TABLE 2:** Action potential parameters in primary atrial myocytes.

Parameter	WT+PBS	WT+FGF23	*P*‐value
RP, mV	−73.98 ± 5.18	−76.53 ± 8.61	0.4335
APA, mV	116.7 ± 6.96	128.8 ± 7.71	0.0017
*V*_max_, mV/ms	151.2 ± 34.95	188.8 ± 44.49	0.0500
APD_20_, ms	2.9 ± 1.44	15.36 ± 3.15	*P <* 0.0001
APD_50_, ms	9.23 ± 5.81	53.83 ± 11.64	*P <* 0.0001
APD_90_, ms	29.79 ± 16.55	107.0 ± 11.75	*P <* 0.0001

Abbreviations: APA, action potential amplitude; APD20, action potential duration at 20% repolarization; APD50, action potential duration at 50% repolarization; APD90, action potential duration at 90% repolarization; FGF23, fibroblast growth factor 23; PBS, phosphate‐buffered saline; RP, resting membrane potential; Vmax, maximum upstroke velocity; WT, wild‐type.

Moreover, having elucidated the impact of FGF23 on *I*_Na_ in adult murine atrial myocytes, we investigated its effects on sodium current properties further in primary atrial myocytes from neonatal mice. The results demonstrated that the FGF23‐treated group exhibited an upward shift in *I*_Na_ density at −20 mV compared with the control group, along with a significant increase in peak *I*_Na_ density amplitude (−312.1 ± 54.44 vs. −389.5 ± 59.67 pA/pF, *P* < 0.01; Figure 5a–c). Furthermore, although no significant differences were observed in SSA between the two groups, the SSI curve in the FGF23‐treated group shifted rightwards, with a significantly increased *V*_1/2, inact_ (−74.41 ± 5.51 vs. −65.19 ± 6.67 mV, *P* < 0.01) and no notable change in *K*_inact_, collectively indicating delayed sodium channel inactivation (Figure 5d–g). Additionally, the residual sodium current measured 3 ms after peak *I*_Na_ was significantly greater in FGF23‐treated neonatal mouse atrial myocytes than in control cells [WT+PBS vs. WT+FGF23: 6.71% (5.27%–10.34%) vs. 17.54% (11.65%–26.27%), *P* < 0.05; Figure 5h, i]. Western blot analysis confirmed a marked upregulation of Nav1.5 protein expression after FGF23 treatment (Figure 5j, k). Subsequent superposition of SSA and inactivation curves revealed an enhanced window current following FGF23 treatment (Figure 5l).

**FIGURE 5:**
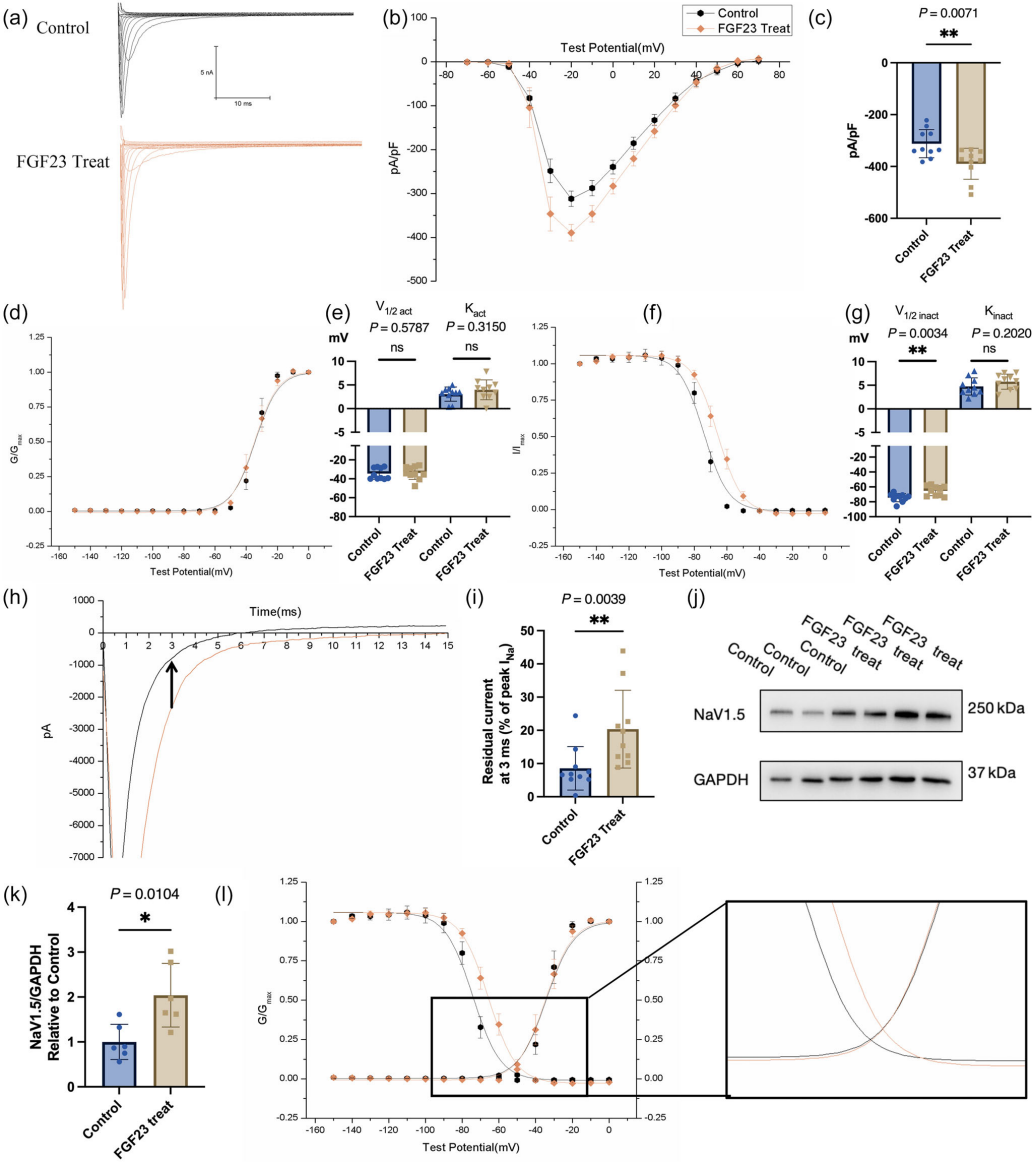
Effects of FGF23 stimulation on *I*_Na_ and electrophysiological properties in primary atrial myocytes. (a) Typical *I*_Na_ trajectories of primary atrial myocytes in the voltage‐clamp mode. (b, c) *I–V* curve (b) and quantitative analysis of peak sodium current density at −20 mV (c) in control and FGF23 groups. *n* = 10 cells from six mice. (d, e) *I*_Na_ steady‐state activation curves (d) and parameter statistics (e) in control and FGF23 group. *n* = 10. (f, g) Steady‐state inactivation curves (f) and parameter statistics (g) in acutely isolated mouse atrial myocytes. *n* = 10 cells from six mice. (h) Residual sodium current measured 3 ms after peak *I*_Na_ at a test potential of −20 mV in primary atrial myocytes from both groups (black arrow indicates the current measured 3 ms after the peak current). (i) Quantitative analysis of residual sodium current measured 3ms after peak *I*_Na_, expressed as a percentage of peak *I*_Na_, *n* = 10 cells from 6 mice. (j, k) Representative western blotting (j) and quantitative analysis of Nav1.5 proteins (k). *n* = 6. (l) Window currents of sodium channels (black line indicates control group; yellow line indicates FGF23 group). ns, not significant, *P < 0.05 and **P < 0.01. Abbreviations: *I*_Na_, sodium current; NaV1.5, voltage‐gated sodium channel 1.5.

### 3.4 FGF23 stimulation disrupts intracellular calcium regulation in adult mouse atrial myocytes

The aforementioned results demonstrated that FGF23 stimulation prolongs APD and increases the incidence of DADs in atrial myocytes; a process closely associated with intracellular calcium handling. Therefore, we further investigated the effects of FGF23 on intracellular calcium regulation. Compared with the control group, atrial myocytes from FGF23‐treated mice exhibited a significantly larger amplitude of L‐type calcium current (I_Ca,L_; −7.24 ± 0.52 vs. −10.46 ± 1.03 pA/pF, *P* < 0.05; Figure 6a–c), although no significant differences were observed in the SSA or inactivation curves of *I*_Ca,L_ (Figure 6d,e,g,h). Further analysis of the time constant of *I*_Ca,L_ recovery from inactivation (τ) revealed a significant decrease following FGF23 stimulation (298.5 ± 25.37 vs. 219.4 ± 26.58 ms, *P* < 0.05; Figure 6f,i). These findings suggest that FGF23 might enhance *I*_Ca,L_ by altering the kinetics of recovery from inactivation rather than modifying steady‐state gating properties, thereby increasing the frequency of calcium channel reopening.

**FIGURE 6:**
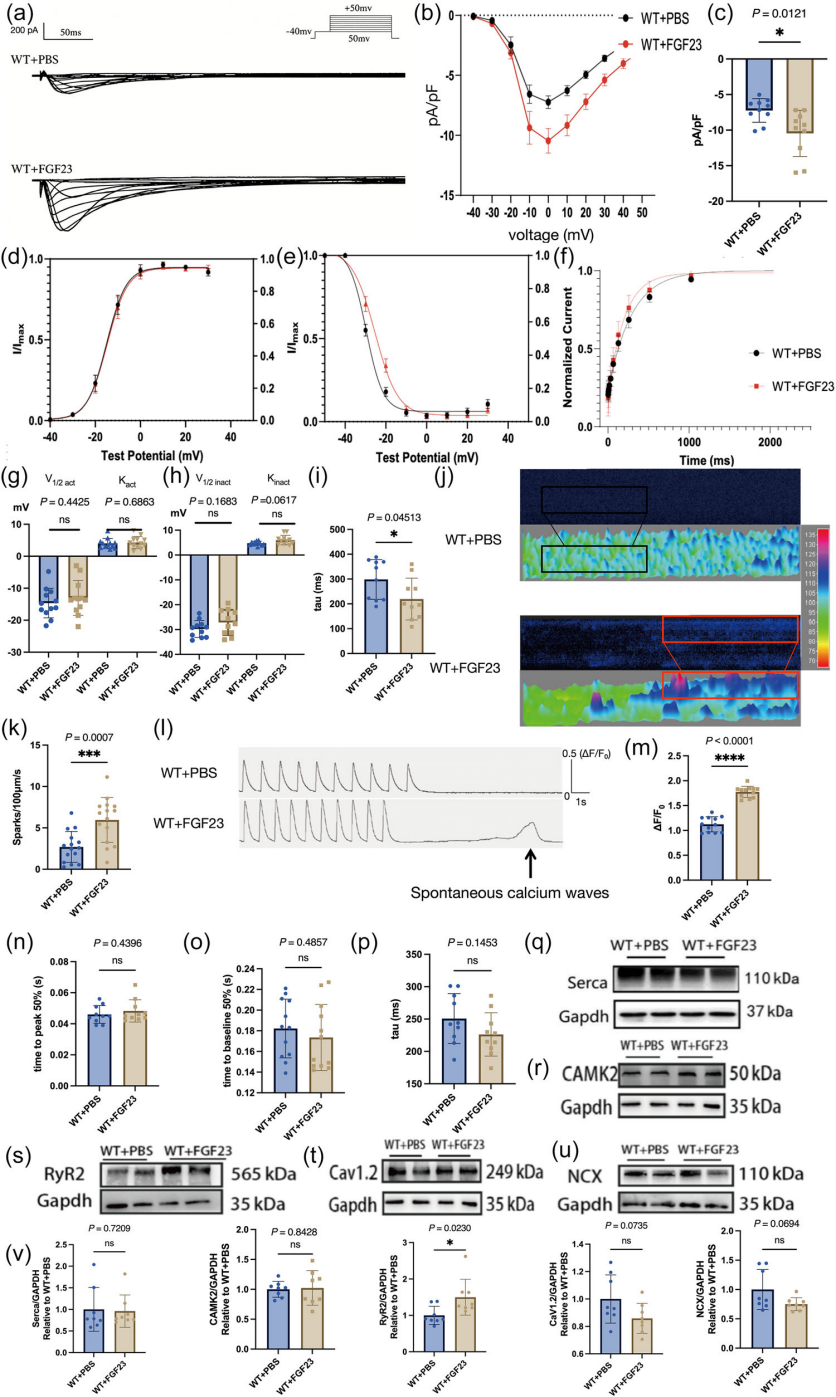
Regulation of intracellular calcium homeostasis by FGF23 in adult mouse atrial myocytes. (a) Typical *I*_Ca,L_ trajectories of acutely isolated mouse atrial myocytes in voltage‐clamp mode, with stimulation procedure on the right. (b–c) *I–V* curve (b) and quantitative analysis of peak calcium current density (*I*_Ca,L_) at 0 mV in WT+PBS and WT+FGF23 group (c). *n* = 10 cells from six mice. (d–i) Steady‐state gating and recovery kinetics of *I*_Ca_: (d) activation; (e) inactivation; and (f) recovery curves of *I*_Ca_. Corresponding parameter statistics are shown as follows: (g) *V*_1/2 act_ and *K*_act_; (h) V_1/2 inact_ and *K*_inact,_; and (i) recovery τ, *n* = 10–12 cells from six mice. (j) Representative images of calcium spark captured using confocal microscopy in the WT+FGF23 and WT+PBS groups. The black rectangles represent regions with fewer calcium sparks, appearing as flat plains in the 3D images, and the red rectangles represent regions with more calcium sparks, appearing as clustered peaks in the 3D images. (k) Quantitative analysis of calcium spark frequency in each group. *n* = 15 cells from eight mice. (l) The cardiomyocytes of WT+FGF23 and WT+PBS groups were stimulated at 1 Hz and recorded for 10 s after the stimulation. The arrow indicates the occurrence of spontaneous calcium waves. (m) Quantitative analysis of the amplitude of calcium transients in each group. *n* = 12 cells from six mice. (n–p) Quantitative analysis of time to peak (n), time to 50% decay (o) and tau (τ) of calcium decay (p) in each group. *n* = 10–12 cells from six mice. (q–v) Representative western blotting and quantitative analysis of SERCA2a, CAMKII, RyR2, Cav1.2 and NCX1 proteins in each group. *n* = 8. ns, not significant, *P < 0.05, **P < 0.01, ***P < 0.001 and ****P < 0.0001. Abbreviations: CaMKII, calcium/calmodulin‐dependent protein kinase II; CaV1.2, voltage‐gated calcium channel 1.2; *I*_Ca_, calcium current; *I*_Ca,L_, L‐type calcium current; NCX, sodium‐calcium exchanger; PBS, phosphate‐buffered saline; RyR2, ryanodine receptor 2; SERCA2a, sarco/endoplasmic reticulum Ca²⁺‐ATPase 2a; WT, wild‐type.

Furthermore, the frequency of diastolic calcium sparks was significantly elevated in FGF23‐treated myocytes compared with control myocytes (5.96 ± 2.71 vs. 2.70 ± 1.87 sparks/100 µm/s, *P* < 0.001; Figure 6j,k), indicating that FGF23 might induce intracellular calcium overload by promoting SR calcium leakage. Subsequently, we examined whether FGF23 influences intracellular calcium transients or reuptake processes in murine atrial myocytes. The results showed that the calcium transient amplitude was significantly higher in the FGF23 group than in the control group (1.78 ± 0.11 vs. 1.12 ± 0.15, *P* < 0.0001), and spontaneous calcium waves were observed (Figure 6l,m), indicating that FGF23 disrupts intracellular calcium handling and might increase SR calcium release through enhanced RyR2 activity and/or altered SR calcium loading. No significant differences were detected between the FGF23 and control groups in the time to 50% peak, half‐decay time or time constant (τ) of calcium decay (Figure 6n–p), suggesting that FGF23 primarily affects the magnitude of calcium transients without markedly altering the kinetics of calcium release or reuptake (e.g., the rising and falling rates of calcium transients).

Beyond assessing channel activity, we also evaluated the expression of key calcium‐handling proteins. FGF23 was found to upregulate the expression of RyR2 in mouse atrial myocytes, whereas no significant changes were observed in the protein levels of Cav1.2, NCX1, SERCA2a or CAMKII (Figure 6q–v).

### 3.5 Regulation of intracellular calcium handling by FGF23 in primary atrial myocytes

To exclude potential confounding effects caused by plsystemic alterations in phosphate metabolism or other factors following tail vein injection of FGF23, we conducted in vitro experiments to examine the direct influence of FGF23 on calcium handling in atrial myocytes. Our results demonstrated that FGF23 treatment significantly enhanced *I*_Ca,L_ in neonatal mouse atrial myocytes (−16.54 ± 1.47 vs. −9.22 ± 1.24 pA/pF, *P* < 0.01; Figure 7a–c). No significant changes were observed in the SSA or inactivation properties of *I*_Ca,L_. However, the kinetics of recovery from inactivation were accelerated, as indicated by a decreased time constant (138.3 ± 16.77 vs. 214.1 ± 28.32 ms, *P* < 0.001; Figure 7d–i), suggesting facilitated recovery of L‐type calcium channels from inactivation; a result consistent with our in vivo findings. Given the relative immaturity of the SR in neonatal mouse atrial myocytes, which limits the robustness of calcium transient and calcium spark assays for characterizing intracellular calcium metabolism, we focused on evaluating the expression of key calcium‐handling proteins. In agreement with previous observations, FGF23 treatment upregulated the expression of RyR2, whereas no significant differences were detected in the protein levels of Cav1.2, NCX1, SERCA2a or CAMKII (Figure 7j–o).

**FIGURE 7:**
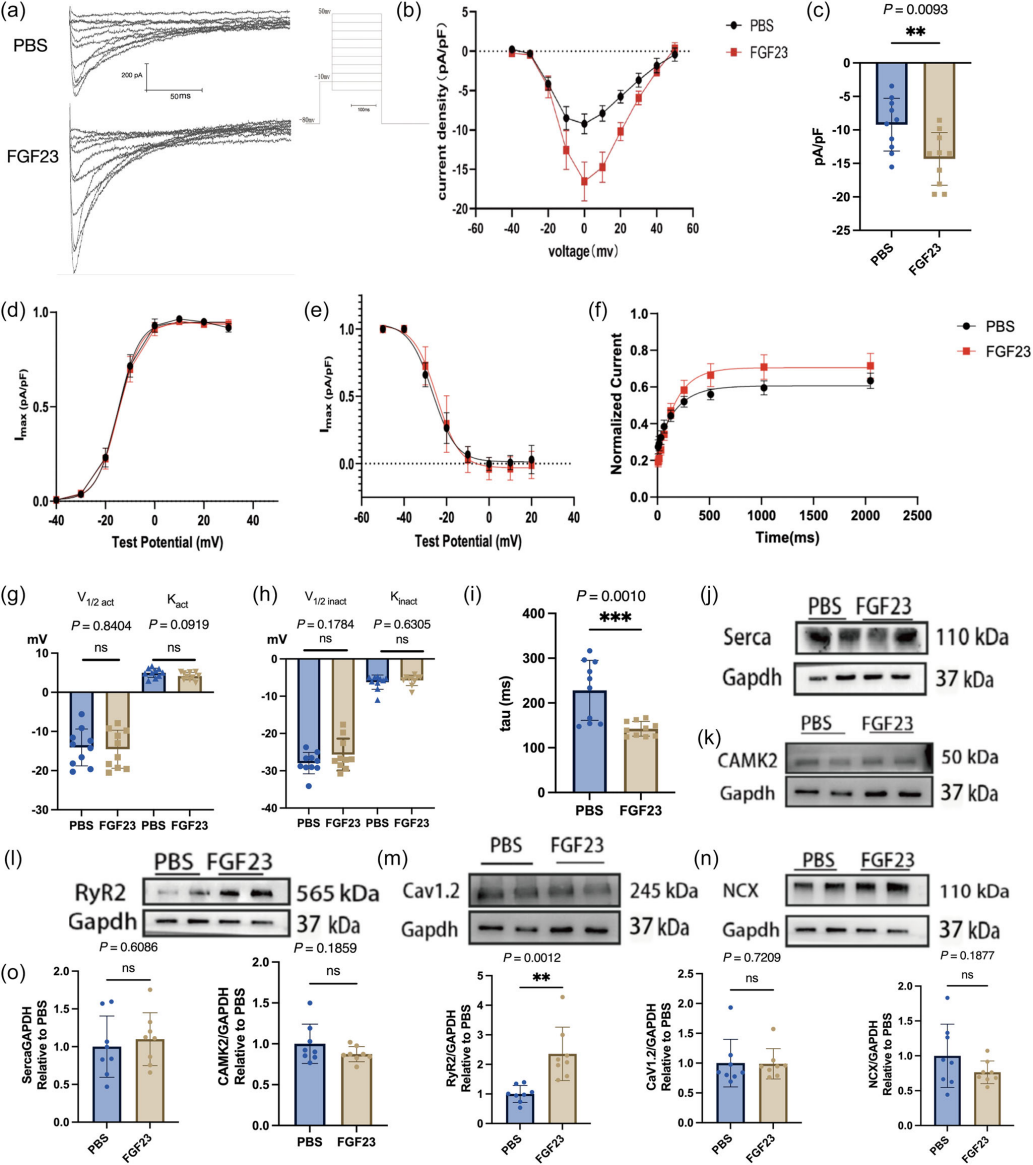
Regulation of intracellular calcium by FGF23 in primary atrial myocytes from neonatal mice. (a) Typical *I*_Ca,L_ trajectories of primary atrial myocytes in voltage‐clamp mode, with stimulation procedure on the right. (b, c) *I–V* curve (b) and quantitative analysis of peak calcium current density (*I*_Ca,L_) at 0 mV in PBS and FGF23 groups (c). *n* = 10 cells from six mice. (d–i) Steady‐state gating and recovery kinetics of *I*_Ca_: (d) activation; (e) inactivation; and (f) recovery curves of *I*_Ca_. Corresponding parameter statistics are shown as follows: (g) *V*_1/2 act_ and *K*_act_; (h) *V*_1/2 inact_ and K_inact_; and (i) recovery τ, *n* = 10 cells from six mice. (j–o) Representative western blotting and quantitative analysis of SERCA2a, CAMKII, RyR2, Cav1.2 and NCX1 proteins in each group. *n* = 8. ns, not significant, *P < 0.05, **P < 0.01 and ***P < 0.001. Abbreviations: CaMKII, calcium/calmodulin‐dependent protein kinase II; CaV1.2, voltage‐gated calcium channel 1.2; *I*_Ca_, calcium current; *I*_Ca,L_, L‐type calcium current; NCX, sodium‐calcium exchanger; PBS, phosphate‐buffered saline; RyR2, ryanodine receptor 2; SERCA2a, sarco/endoplasmic reticulum Ca²⁺‐ATPase 2a.

### 3.6 ETV1 might act as a downstream effector molecule mediating the actions of FGF23

To investigate the functional interplay between FGF23 and ETV1 in regulating sodium current and action potentials in atrial myocytes, we performed both knockdown and overexpression of ETV1 in primary cultured cells followed by FGF23 treatment. In comparison to the ETV1‐NC+PBS group, the ETV1‐Si+PBS group exhibited significantly reduced APA and *V*_max_, whereas no significant differences were observed in APD or RP. Conversely, the ETV1‐OE+PBS group showed markedly increased APA and *V*_max_ without significant changes in RP or APD (Figure 8a–m). In ETV1 knockdown cells, FGF23 significantly increased APA, but did not significantly affect RP or *V*_max_. In ETV1‐overexpressing cells, FGF23 significantly reduced APA and *V*_max_, whereas RP remained unchanged (Figure 8a–g). Notably, FGF23 treatment significantly prolonged APD regardless of ETV1 manipulation (knockdown or overexpression; Figure 8h–m).

**FIGURE 8:**
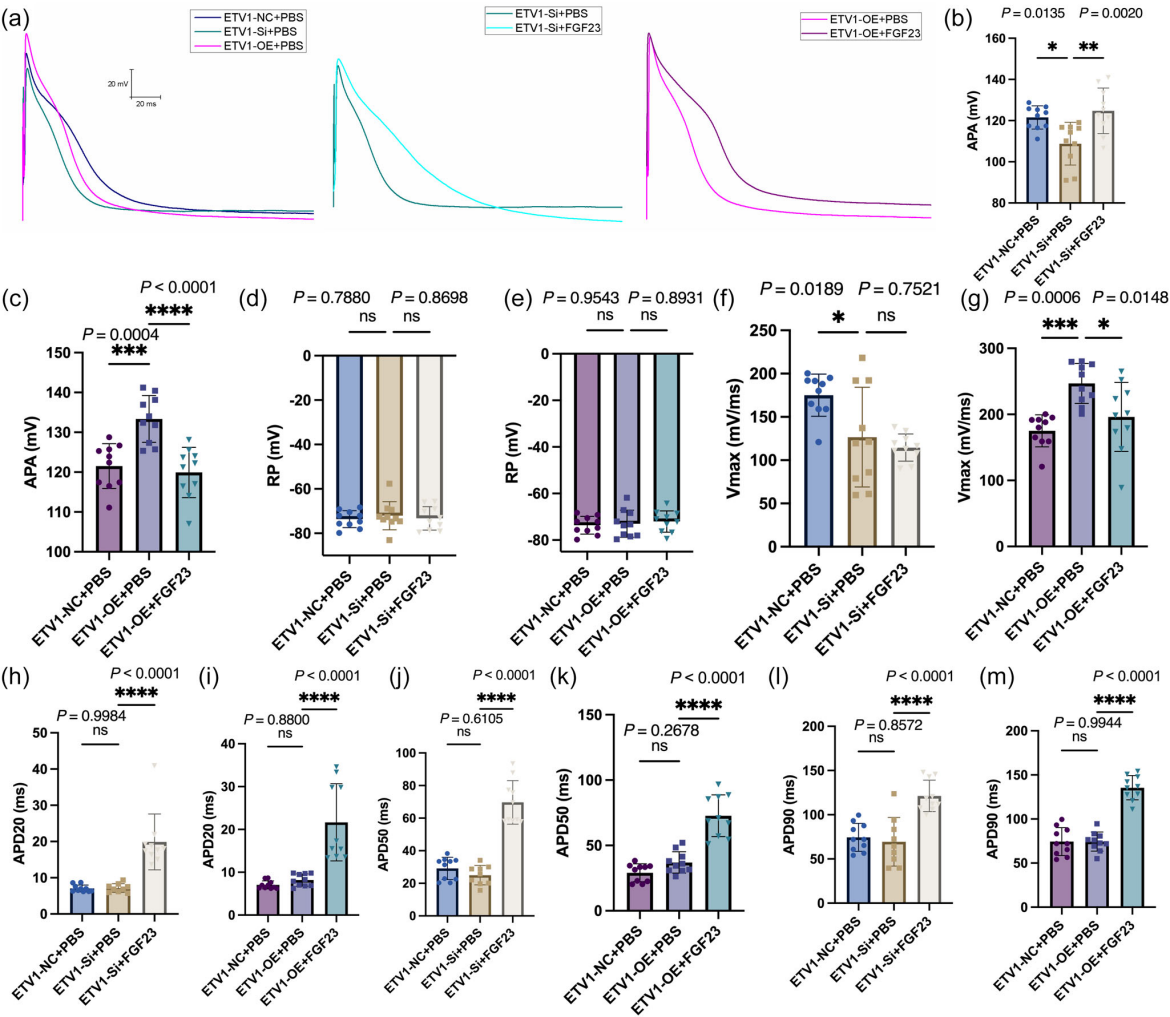
Effects of ETV1 on action potential in primary atrial myocytes. (a) Typical action potential trajectories in each group. (b–m) Quantitative analysis of APA, RP, *V*_max_, APD_20_, APD_50_ and APD_90_ in each group. *n* = 9–10 cells from six mice. ns, not significant, *P < 0.05, **P < 0.01, ***P < 0.001 and ****P < 0.0001. Abbreviations: APA, action potential amplitude; APD20, action potential duration at 20% repolarization; APD50, action potential duration at 50% repolarization; APD90, action potential duration at 90% repolarization; NC, negative control; OE, overexpression; PBS, phosphate‐buffered saline; RP, resting membrane potential; si, small interfering RNA; Vmax, maximum upstroke velocity.

ETV1 knockdown significantly decreased peak sodium current density, whereas ETV1 overexpression increased it (Figure 9a–c). However, no further modulation of peak sodium current was observed upon FGF23 treatment in either ETV1 knockdown or overexpressing cells (Figure 9a–c). Given that FGF23 alone enhanced peak *I*_Na_ but failed to alter it upon ETV1 manipulation, these results suggest that ETV1 might serve as a key downstream effector mediating the effects of FGF23.

**FIGURE 9:**
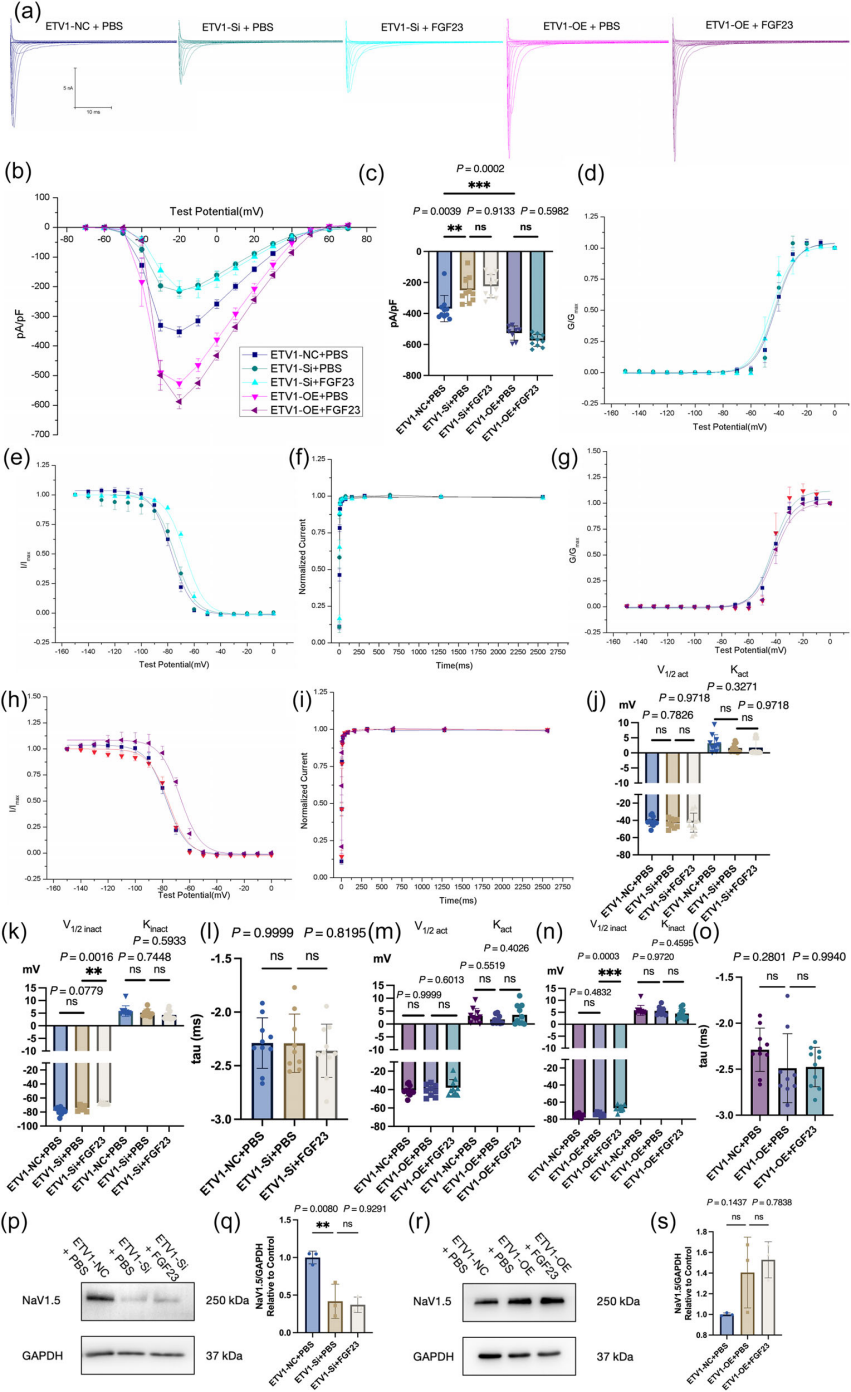
Effects of ETV1 on *I*_Na_ in primary atrial myocytes. (a) Typical *I*_Na_ trajectories of primary atrial myocytes in the voltage‐clamp mode in each group. (b, c) *I–V* curve (b) and quantitative analysis of peak sodium current density at −20 mV in each group (c). *n* = 8–10 cells from six mice. (d–o) Steady‐state gating and recovery kinetics of *I*_Na_: (d, g) activation; (e, h) inactivation; and (f, i) recovery curves of *I*_Na_ in each group (d–f, knockdown; g–i, overexpression). Corresponding parameter statistics are shown as follows: (j, m) *V*_1/2 act_ and *K*_act_; (k, n) *V*_1/2 inact_ and *K*_inact_; and (l, o) recovery τ. (j–l, knockdown; m–o, overexpression), *n* = 8–10 cells from six mice. (p–s) Representative western blotting and quantitative analysis of Nav1.5 proteins in each group. *n* = 3. ns, not significant, *P < 0.05, **P < 0.01 and ***P < 0.001. Abbreviations: *I*_Na_, sodium current; NaV1.5, voltage‐gated sodium channel 1.5; NC, negative control; OE, overexpression; PBS, phosphate‐buffered saline; Si, small interfering RNA.

We analysed sodium channel gating properties further. ETV1 knockdown alone did not alter SSA or inactivation curves of *I*_Na_. Although SSA remained unchanged, the SSI curve showed a rightward shift with a significantly increased *V*_1/2, inact_ in the ETV1 knockdown+FGF23 group (Figure 9d,e,j,k). No significant differences were detected in recovery from inactivation among groups (Figure 9f,l). Likewise, ETV1 overexpression alone did not affect SSA or inactivation, but the addition of FGF23 induced a rightward shift of the inactivation curve and an increase in *V*_1/2, inact_ (Figure 9g,h,m,n). Recovery from inactivation remained unaltered across all ETV1‐overexpressing groups (Figure 9i,o). Furthermore, ETV1 knockdown significantly reduced Nav1.5 protein expression in neonatal mouse atrial myocytes. FGF23 treatment failed to increase Nav1.5 expression in ETV1‐deficient cells. In contrast, ETV1 overexpression did not increase Nav1.5 protein expression further, and FGF23 treatment produced no additional effect in ETV1‐overexpressing cells (Figure 9p–s).

### 3.7 Effects of ETV1 knockout and FGF23 stimulation on susceptibility to AF

We generated ETV1 knockout (ETV1‐KO) mice to investigate the susceptibility to AF, impact on *I*_Na_, atrial myocyte electrophysiological properties, and its interplay with FGF23. We assessed whether ETV1 knockout and FGF23 stimulation modulate AF susceptibility. Both FGF23 injection and ETV1 knockout increased AF inducibility compared with WT control animals. However, no significant difference in AF inducibility was observed between FGF23‐injected WT mice and ETV1‐KO mice (Figure 10a,b; Table 3). Likewise, AF duration did not differ significantly among the groups (Figure 10c; Table 3). Compared with the control group, ETV1‐KO atrial myocytes exhibited significantly reduced APA and *V*_max_, elevated RP and no significant change in APD (Figure 10d–j; Table 4). Relative to the ETV1‐KO+PBS group, the ETV1‐KO+FGF23 group showed no differences in RP, APA or *V*_max_ but demonstrated prolonged APD (Figure 10d–j; Table 4).

**FIGURE 10:**
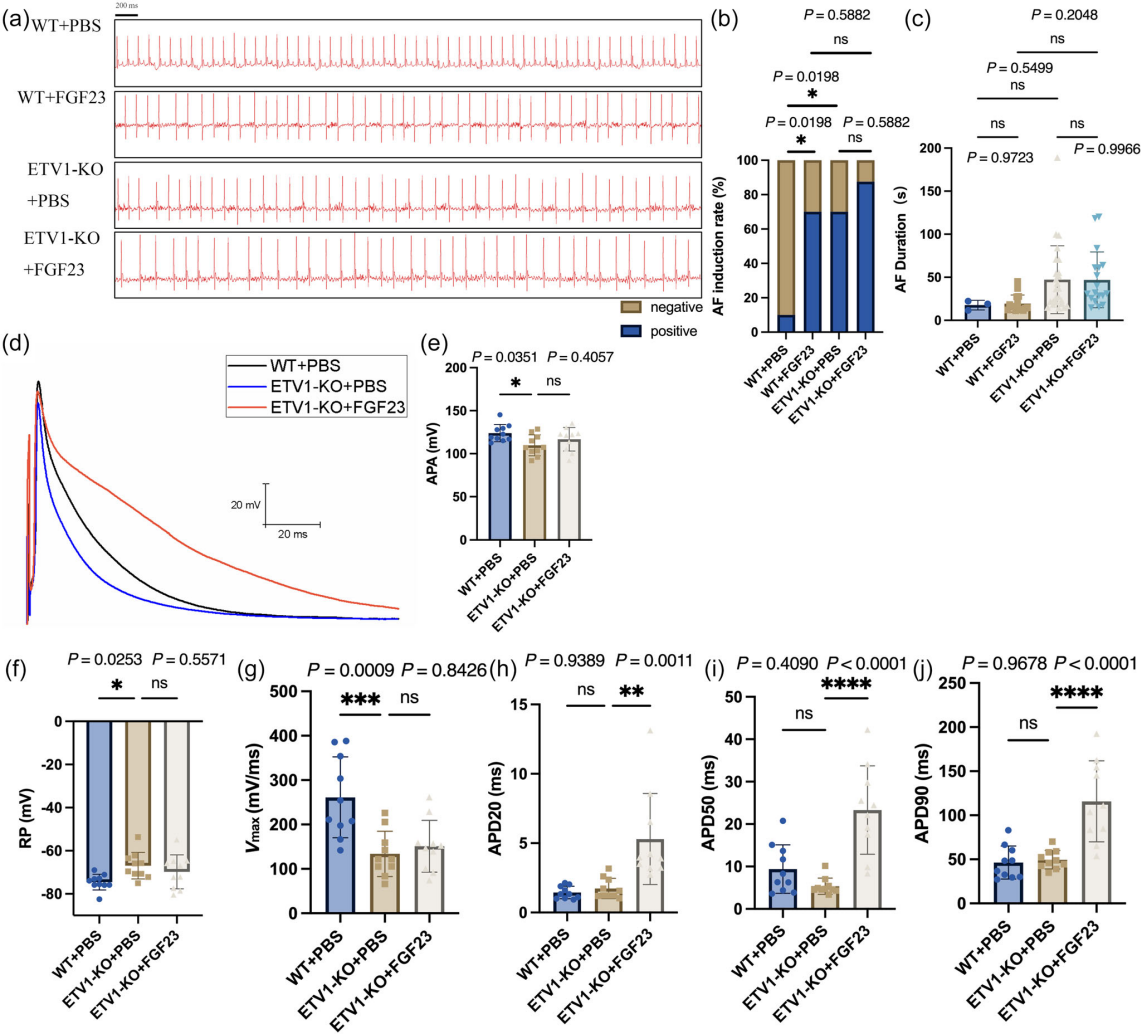
Effects of ETV1 knockout and FGF23 stimulation on AF susceptibility and action potential properties. (a) Representative surface ECG traces illustrating AF induction after atrial pacing in each group. (b, c) Statistics of transoesophageal AF induction rate and duration of AF in mice, *n* = 8–10. (d) Typical action potential trajectories in each group. (e–j) Quantitative analysis of APA, RP, *V*_max_, APD_20_, APD_50_ and APD_90_ in each group. *n* = 10 cells from six mice. ns, not significant, *P < 0.05, **P < 0.01, ***P < 0.001 and ****P < 0.0001. Abbreviations: AF, atrial fibrillation; APA, action potential amplitude; APD20, action potential duration at 20% repolarization; APD50, action potential duration at 50% repolarization; APD90, action potential duration at 90% repolarization; ECG, electrocardiogram; KO, knockout; PBS, phosphate‐buffered saline; RP, resting membrane potential; Vmax, maximum upstroke velocity.

**TABLE 3:** Induction rate and duration of atrial fibrillation in mice.

Parameter	WT +PBS	WT +FGF23	ETV1‐KO +PBS	ETV1‐KO +FGF23
*n*	10	10	10	8
AF incidence, %	10(1/10)	70(7/10)	70(7/10)	87.5(7/8)
AF duration, s	19.11 (11.58, 22.33)	15.87 (12.27, 25.46)	36.64 (20.43, 62.95)	31.46 (24.64, 61.84)

Abbreviations: AF, atrial fibrillation; FGF23, fibroblast growth factor 23; KO, knockout; PBS, phosphate‐buffered saline; WT, wild‐type.

**TABLE 4:** Action potential parameters in ETV1‐KO mice.

Parameter	WT+PBS	ETV1‐KO+PBS	ETV1‐KO+FGF23
RP, mV	−74.56 ± 3.61	−66.92 ± 6.16	−69.78 ± 7.87
APA, mV	123.9 ± 9.97	109.7 ± 12.16	116.7 ± 13.68
*V*_max_, mV/ms	261.1 ± 91.04	133.8 ± 51.00	151.0 ± 58.20
APD_20_, ms	1.44 ± 0.45	1.400 (1.225, 2.425)	3.933 (3.225, 7.042)
APD_50_, ms	9.40 ± 5.73	4.667 (4.483, 5.825)	23.32 ± 10.42
APD_90_, ms	46.33 ± 18.72	49.52 ± 10.55	115.8 ± 45.99

Abbreviations: APA, action potential amplitude; APD20, action potential duration at 20% repolarization; APD50, action potential duration at 50% repolarization; APD90, action potential duration at 90% repolarization; FGF23, fibroblast growth factor 23; KO, knockout; PBS, phosphate‐buffered saline; RP, resting membrane potential; Vmax, maximum upstroke velocity; WT, wild‐type.

Further analysis of *I*_Na_ revealed a significant reduction in peak sodium current density in ETV1‐KO mice compared with control animals (Figure 11a–c). No significant differences were observed in the SSA, SSI or recovery time constant (τ_1_) of *I*_Na_ between WT+PBS and ETV1‐KO+PBS (Figure 11d–i). The window current remained comparable between groups (Figure 11l). In contrast, the ETV1‐KO+FGF23 group exhibited a more pronounced rightward shift in the SSI curve compared with the SSA curve relative to the ETV1‐KO+PBS group (Figure 11d–g), with significant changes in both half‐activation and half‐inactivation voltages (*V*_1/2, act_, −52.14 ± 12.63 vs. −36.61 ± 14.15 mV, *P* < 0.05; *V*_1/2, inact_, −69.68 ± 4.50 vs. −51.32 ± 10.19 mV, *P* < 0.001). Consequently, superposition of the SSA and inactivation curves indicated a significantly larger window current in the ETV1‐KO+FGF23 group than in the ETV1‐KO+PBS group (Figure 11l). No significant differences were detected in recovery kinetics or τ_1_ between two groups (Figure 11h,i). Additionally, Nav1.5 protein expression was significantly downregulated in atrial tissues from ETV1‐KO mice compared with wild‐type (WT) mice. Moreover, FGF23 treatment failed to upregulate Nav1.5 expression in ETV1‐KO mice relative to the ETV1‐KO+PBS group (Figure 11j,k).

**FIGURE 11:**
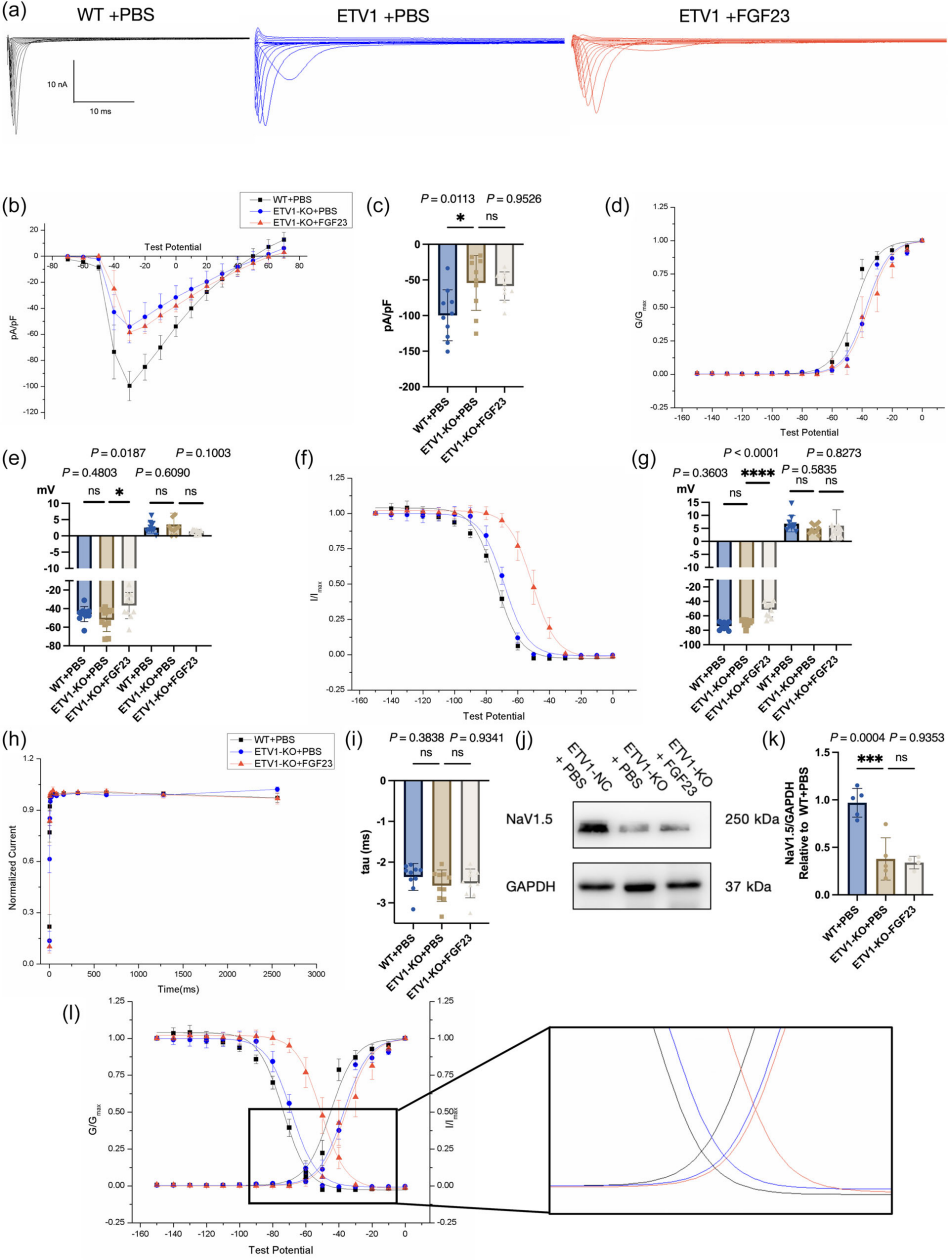
Effects of ETV1 knockout and FGF23 stimulation on sodium channel properties and Nav1.5 expression. (a) Typical *I*_Na_ trajectories in the voltage‐clamp mode in acutely isolated mouse atrial myocytes of WT+PBS, ETV1‐KO+PBS and ETV1‐KO+FGF23 mice. (b, c) *I–V* curve (b) and quantitative analysis of peak sodium current density at −20 mV in each group (c). *n* = 10 cells from six mice. (d, e) *I*_Na_ steady‐state activation curves (b) and parameter statistics (c) in acutely isolated mouse atrial myocytes in each group. *n* = 10 cells from six mice. (f, g) Steady‐state inactivation curves (f) and parameter statistics (g) in acutely isolated mouse atrial myocytes in each group. *n* = 10 cells from six mice. (h, i) Recovery kinetic curves (h) and parameter statistics (i) in each group. *n* = 10 cells from six mice. (j, k) Representative western blotting (j) and quantitative analysis of Nav1.5 proteins (k) in each group. (l) Window currents of sodium channels (black line indicates WT+PBS group; blue line indicates ETV1‐KO+PBS group; and red line indicates ETV1‐KO+FGF23 group). ns, not significant, *P < 0.05, **P < 0.01, ***P < 0.001 and ****P < 0.0001. Abbreviations: KO, knockout; PBS, phosphate‐buffered saline.

## 4 DISCUSSION

Our findings demonstrate that elevated circulating FGF23 enhances *I*_Na, peak_ and window sodium current and increases the residual sodium current measured shortly after peak current activation. It also increases *I*_Ca, L_ density, augments cytosolic calcium transient amplitude, elevates calcium spark frequency and promotes SR calcium leak. These perturbations collectively prolong APD, increase the incidence of EADs and DADs, enhance triggered activity and, ultimately, promote AF. Likewise, ETV1‐deficient mice exhibited increased AF susceptibility, reduced sodium current, downregulated Nav1.5 expression and decreased APA and *V*_max_, resulting in slowed conduction velocity and increased micro‐reentry circuits. These observations suggest that ETV1 might contribute to FGF23‐mediated regulation of Nav1.5 expression and sodium channel function.

FGF23 increased both *I*_Na, peak_ and APA, and the upregulation of Nav1.5 expression might contribute to these changes. Enlargement of the sodium window current during FGF23 stimulation might increase persistent sodium influx during the early phase of repolarization, thereby contributing to APD prolongation. It also maintains elevated depolarizing drive during repolarization, facilitating EAD formation. Interestingly, although EADs were not observed in adult murine atrial myocytes, they were evident in primary neonatal cells, possibly owing to the lower differentiation status and greater membrane sensitivity of neonatal cells to membrane depolarization, rendering them more prone to triggered activity. Furthermore, this study elucidates the role of ETV1 in regulating sodium current. Prior work reported that cardiac‐specific knockout of ETV1 promotes SSI of sodium channels at more negative potentials and slows recovery from inactivation (Yamaguchi et al., 2021). Likewise, we found that ETV1 knockout reduces Nav1.5 expression and *I*_Na, peak_, albeit without significant changes in gating kinetics; a discrepancy that might be related to differences in experimental models and study conditions, because the present study used a global ETV1 knockout model, whereas Yamaguchi et al. (2021) took a cardiac‐specific knockout approach. Nonetheless, ETV1 deficiency consistently reduces sodium current, leading to decreased APA and *V*_max_. These reductions slow action potential conduction and impair frequency adaptation, thereby facilitating multiple‐wavelet reentry and promoting AF initiation and maintenance. Finally, we investigated whether ETV1 contributes to the sodium channel regulatory effects of FGF23. ETV1 manipulation altered the effects of FGF23 on Nav1.5 expression, *I*_Na, peak_ and *V*_max_, without affecting APD. This indicates that ETV1 is involved in FGF23‐mediated modulation of sodium channel function.

Delayed or incomplete inactivation of the Nav1.5 channel leads to persistent sodium influx, which prolongs the repolarization phase of the action potential; a classic ‘gain‐of‐function’ phenotype (Hegyi et al., 2021; Wilde & Amin, 2018). In contrast, a reduction in peak sodium current might slow cardiac conduction velocity or cause region‐specific repolarization heterogeneity, representing a ‘loss‐of‐function’ phenotype (Antzelevitch et al., 2002; Krahn et al., 2022). By comparing these two phenotypes, the essential role of Nav1.5 in cardiac electrophysiology becomes evident. Maintaining normal Nav1.5 function is crucial for proper repolarization and conduction in the heart, and any dysregulation might disrupt electrical synchrony, potentially leading to arrhythmias such as AF. Therefore, elucidation of the regulatory mechanisms of Nav1.5 and its relationship with cardiac electrophysiology is of great significance for the prevention and treatment of cardiac diseases. In this study, we found that FGF23 induces an enhanced window current, consistent with a gain‐of‐function phenotype, whereas ETV1 deficiency results in a loss‐of‐function phenotype. Together, these findings support distinct roles of FGF23 and ETV1 in regulating atrial sodium channel function.

In addition to its modulation of sodium handling, we found that FGF23 enhances intracellular calcium transients and increases calcium spark frequency without affecting the time to peak or decay kinetics of calcium transients. The upregulation of RyR2 expression provides a molecular basis for the increased calcium transient amplitude, because RyR2 is primarily responsible for SR calcium release during systole. In contrast, SERCA2a and NCX1 (key regulators of diastolic calcium reuptake and extrusion) remained unchanged, explaining why FGF23 did not alter calcium decay kinetics. Ca^2^^+^/calmodulin‐dependent protein kinase II (CaMKII) is a pivotal signalling enzyme that regulates calcium homeostasis in cardiomyocytes (Gui et al., 2018). Although total CaMKII expression was unchanged in the present study, Kao et al. (2014) reported increased phosphorylation of CaMKII and phospholamban (PLB) following FGF23 stimulation. Therefore, a contribution of CaMKII‐dependent signalling cannot be excluded but was not directly supported by our protein expression data. The observed increase in calcium spark frequency is likely to be attributable to elevated SR Ca^2+^ leak. Spontaneous calcium release events play a crucial role in AF initiation. For instance, mice deficient in FKBP12.6 (a stabilizing subunit of RyR2) exhibit greater SR Ca^2+^ leak, more frequent spontaneous calcium release events (Ljones et al., 2017), activation of NCX and increased DADs, accompanied by heightened susceptibility to pacing‐induced AF. Similar findings were observed in mice carrying the Junctophilin‐2‐E169K mutation (Lenaerts et al., 2009), which weakens RyR2 interaction, underscoring the determinative role of RyR2 functional integrity in SR calcium leak. Furthermore, in the present study we observed an upregulation of RyR2 protein expression following FGF23 treatment, suggesting that FGF23 might enhance its open probability through direct transcriptional or translational regulation rather than solely via post‐translational modification. However, previous in vitro studies by Navarro‐García et al. (2019, 2020) reported unchanged RyR2 expression but increased RyR2 phosphorylation in FGF23‐stimulated cardiomyocytes. This discrepancy might be attributed to differences in FGF23 concentration and exposure duration, indicating that its effects might be both dose and time dependent. A limitation of our study is the lack of direct assessment of RyR2 phosphorylation status. Although SR Ca^2+^ content and NCX activity were not measured directly in the present study, previous studies provide supportive evidence for our interpretation. FGF23 exposure in mouse atrial myocytes and rabbit pulmonary vein myocytes has been reported to disrupt intracellular Ca^2+^ homeostasis, including enhanced Ca^2+^ leak and altered NCX‐mediated exchange currents (Huang et al., 2016; Kao et al., 2014). Furthermore, a recent study demonstrated that cardiac FGF23 overexpression in mouse atrial myocytes is associated with increased Ca^2+^ leak and enhanced NCX function (Li et al., 2025), supporting a role for FGF23 in regulating intracellular Ca^2+^ handling.

Collectively, these findings suggest that RyR2 is a potential downstream effector contributing to FGF23‐induced calcium dysregulation by amplifying calcium transient amplitude and promoting SR calcium leak, ultimately leading to intracellular calcium overload. In contrast, the enhanced *I*_Ca,L_ further exacerbates intracellular calcium loading and, through calcium‐induced calcium release, potentiates RyR2 activation, triggering more substantial calcium release. Huang et al. (2016) also demonstrated that FGF23 increases *I*_Na,L_ in rabbit pulmonary vein cardiomyocytes, which might activate reverse‐mode NCX operation, further elevating intracellular calcium levels (Fowler et al., 2020). The increase in residual sodium current observed in our study might similarly contribute to intracellular sodium loading and thereby facilitate calcium dysregulation. Excess intracellular calcium, in turn, activates forward‐mode NCX, generating a depolarizing transient inward current (*I*_ti_) that underlies DADs. When DADs reach sufficient amplitude to overcome electrotonic load and activate sodium channels, triggered activity occurs (Virág et al., 2009), establishing a functional substrate for AF.

In summary, FGF23 disrupts intracellular calcium homeostasis, leading to APD prolongation and facilitation of DADs. Concurrently, through its modulation of sodium channel function, FGF23 promotes electrical instability and facilitates EAD formation. These combined electrophysiological effects contribute to the formation of a vulnerable substrate conducive to AF. It is noteworthy, however, that FGF23 administration in this study did not induce structural remodelling of the atria or establish a stable reentrant substrate. Consequently, we observed a significant effect of FGF23 on AF inducibility, but no substantial impact on AF duration was detected.

### 4.1 Limitations

In the present study, we used tail vein injection of FGF23 to elevate circulating FGF23 levels, which might not fully recapitulate the effects of long‐term chronic FGF23 exposure on AF susceptibility. In addition, the FGF23 administration protocol used in this study represents an acute exposure model. Given the relatively short circulating half‐life of FGF23, intermittent tail vein injection might result in transient supraphysiological circulating levels compared with those observed in patients with AF. Although we observed significant alterations in sodium and calcium homeostasis following FGF23 administration, the specific signalling mechanisms underlying these effects remain incompletely elucidated. As a secreted protein, FGF23 might exert its effects by binding to receptors on the atrial myocyte membrane, thereby activating downstream signalling cascades that modulate ETV1 expression or directly influence sodium and calcium channel function. However, we did not investigate the specific receptor interactions or signal transduction pathways involved. Moreover, only male mice were included in this study; therefore, the potential influence of sex differences on the observed findings cannot be excluded. Furthermore, all experiments were conducted in rodent‐based models, both in vitro and in vivo, without validation in human‐derived cells or clinical samples. The absence of correlative FGF23 measurements in patient serum limits the clinical translatability of our findings. Further studies incorporating human cardiomyocytes and clinical cohorts are warranted to strengthen the pathological and therapeutic relevance of these results.

## 5 CONCLUSION

Both elevated circulating FGF23 and ETV1 deficiency were associated with increased susceptibility to AF, reflecting distinct contributions within a common electrophysiological remodelling process. FGF23 prolongs APD and promotes triggered activity through alterations in sodium channel function and intracellular calcium handling, thereby increasing AF susceptibility. ETV1 knockdown partly attenuated the effects of FGF23 on Nav1.5 channel expression and sodium current; however, it did not reduce AF susceptibility, whereas ETV1 deficiency itself impaired atrial sodium channel function and conduction. These findings suggest that FGF23 promotes AF through combined effects of calcium handling abnormalities and ETV1‐mediated sodium channel regulation, thereby enhancing arrhythmogenic susceptibility. ETV1 contributes to the regulation of Nav1.5 expression and sodium channel function but does not fully account for the broader electrophysiological abnormalities induced by FGF23.

## AUTHOR CONTRIBUTIONS

The majority of the experiments were conducted at the Institute of Geriatric Cardiology, Chinese PLA General Hospital, Beijing, China. A portion of the experiments was conducted at the Clinical Medical Research Center, Fuzhou University Affiliated Provincial Hospital, Fujian Provincial Hospital, Fuzhou, China. Qiaoting Yu: Conceptualization, investigation, formal analysis, data curation, writing—original draft. Linwen Zeng: Investigation, formal analysis, data curation, writing—review & editing. Zhijie Chen: Investigation, formal analysis, data curation, writing—review & editing. Zhuhui Lin: Investigation, data curation, writing—review & editing. Congying Wang: Investigation, writing—review & editing. Lan Wang: Investigation, writing—review & editing. Huixian Fu: Conceptualization, methodology, supervision, writing—review & editing. Jiancheng Zhang: Conceptualization, methodology, supervision, writing—review & editing. All authors contributed to the conception or design of the work and/or the acquisition, analysis or interpretation of data; participated in drafting the manuscript or revising it critically for important intellectual content; approved the final version of the manuscript; agree to be accountable for all aspects of the work; and confirm that all persons designated as authors qualify for authorship and that all those who qualify for authorship are listed.

## CONFLICT OF INTEREST

The authors have declared no conflicts of interest.

## GENERATIVE AI STATEMENT

The authors declare that no generative artificial intelligence (GAI) tools were used in the design of the study, data collection, data analysis, interpretation of results, preparation of figures, writing of the manuscript or revision of the manuscript.

## Data Availability

All data supporting the findings of this study are available within the article.
